# Increasing spatial scale of analysis weakens and can mask the dilution effect for hantaviruses

**DOI:** 10.1371/journal.pgph.0007045

**Published:** 2026-09-08

**Authors:** Ana Laura Vigueras-Galván, Celine Arnathau, Oscar Rico-Chávez, Matthew T. Milholland, María L. Vargas, Gerardo Suzán, Carlos Zambrana-Torrelio, Iván Castro-Arellano, James N. Mills, Maria Eugenia Morales, Heliot Zarza Villanueva, Benjamin Roche, Audrey Arnal

**Affiliations:** 1 Laboratorio de Virología, Departamento de Microbiología e Inmunología, Facultad de Medicina Veterinaria y Zootecnia, Universidad Nacional Autónoma de México, Ciudad de México, México; 2 MIVEGEC, Université de Montpellier, IRD, CNRS, Montpellier, France; 3 Departamento de Etología, Fauna Silvestre y Animales de Laboratorio, Facultad de Medicina Veterinaria y Zootecnia, Universidad Nacional Autónoma de México, Ciudad de México, México; 4 Department of Environmental Science and Technology, College of Agriculture and Natural Resources, University of Maryland, College Park, Maryland, United States of America; 5 George Mason University, Fairfax, Virginia, United States of America; 6 Department of Biology, Texas State University, San Marcos, Texas, United States of America; 7 Population Biology, Ecology, and Evolution Program, Emory University, Atlanta, Georgia, United States of America; 8 Organización Panamericana de la Salud (OPS), Ciudad de México, México; 9 Departamento de Ciencias Ambientales, División de Ciencias Biológicas y de la Salud, Universidad Autónoma Metropolitana Unidad Lerma, Lerma de Villada, Estado de México, México; PLOS: Public Library of Science, UNITED STATES OF AMERICA

## Abstract

Despite its major implications for biodiversity conservation and public health, the generality of a protective effect of biodiversity on zoonotic pathogen transmission (*i.e.*, the dilution effect) remains strongly debated. This controversy may partly arise from the diversity of analytical approaches used, but also from the spatial scales at which studies are conducted. Here, we explicitly test whether increasing the spatial scale of analysis affects the detection of the relationship between hantavirus infection prevalence and rodent species richness, using a global dataset spanning multiple regions. Although the association between species richness and infection prevalence remains negative across all spatial scales, we show that the magnitude of the dilution effect progressively weakens as spatial aggregation increases and can become statistically non-significant at coarse spatial resolutions. These results, which are consistent across world regions, indicate that the dilution effect is likely a general feature of hantavirus–rodent systems, but that its empirical detection is highly sensitive to the spatial scale of analysis. Our findings highlight how large-scale spatial aggregation can mask underlying ecological processes and emphasize the need to carefully match spatial scale to the biological mechanisms under investigation when testing biodiversity–disease relationships. Finally, we discuss the ecological and methodological mechanisms that may obscure dilution effects and outline why scale-explicit approaches are essential for robust inference in biodiversity-disease research.

## Introduction

In the mid-twentieth century, the widespread use of vaccines and antibiotics substantially reduced the burden of many infectious diseases and contributed to optimism about their future control [1]. However, this optimism has since been tempered by the steadily increasing frequency of emerging infections, illustrated by the examples of zoonotic diseases like Ebola virus in West Africa, Zika virus in South America, or, more recently the Severe Acute Respiratory Syndrome Coronavirus 2 (SARS-CoV-2) [2,3]. Moreover, these new threats seem to be closely linked to environmental issues such as climate change, land-use change and forest loss, because most of these emerging pathogens originate from wildlife [2].

To this extent, the potential link between the increased transmission of zoonotic pathogens and the reduction of vertebrate biodiversity clearly calls for investigating the possibility of using biodiversity conservation strategies as a public health opportunity [4–7]. Currently, much research focuses on the dilution effect theory, which suggests that a greater species richness within vertebrate communities (hereafter called hosts) can reduce (dilute) pathogen transmission [8,9] through several mechanisms [10]. First, the addition of non-competent host species, *i.e.,* weakly or not at all involved in pathogen transmission within the ecosystem can theoretically reduce transmission of some pathogen species by decreasing contacts among competent host species [11]. However, a dilution effect can also be detected if the abundance of a species that strongly contributes to pathogen transmission, *i.e.,* a highly competent host, negatively correlates with host species richness [12]. It is nevertheless important to point out that a greater species richness within host communities could also increase pathogen prevalence, *i.e.*, creating an amplification effect, and may be associated with enhanced risk of pathogen emergence [9]. Such amplification could also be associated with increases in pathogen richness in parallel with host species diversity [13].

The generality of dilution and amplification effects therefore remains strongly debated [9]. Empirical evidence suggests that the direction and strength of biodiversity-disease relationships can depend on multiple factors, including the composition and complexity of the vertebrate host communities [14–17]. Dilution and amplification processes may even occur concurrently within the same host-pathogen system [18]. As a result, it remains unclear under which ecological contexts dilution or amplification effects should be expected, and which mechanisms primarily drive these contrasting outcomes.

Part of this ongoing debate may stem from methodological heterogeneity among studies, particularly differences in spatial scale, ecosystem type, and metrics used to quantify pathogen transmission [19–23]. Spatial scale is especially critical as ecological processes influencing host community structure and pathogen transmission occur at different spatial scales. Consequently, analyses conducted at mismatched or overly coarse spatial resolutions may obscure underlying biodiversity-disease relationships, contributing to apparently contrasting empirical results. Recent meta-analyses have highlighted the need to disentangle the relative influence of these factors when assessing the generality of dilution or amplification effects [15,17–19,24].

In this study, we test whether the relationship between host species richness and pathogen prevalence—negative under a dilution effect or positive under an amplification effect—depends on the spatial scale at which it is assessed. Beyond the direction of this relationship, our central focus is on its magnitude, i.e., on how strongly an additional rodent species reduces hantavirus prevalence, and on whether that magnitude is preserved as the unit of analysis is coarsened. Using a global scale-explicit re-analysis of a compiled dataset of hantavirus prevalence across rodent host communities, we quantify how systematic changes in the spatial extent of study areas modify the inferred relationship between biodiversity and pathogen prevalence. We specifically examine whether increasing spatial aggregation alters both the strength and the statistical detectability of biodiversity–disease relationships. Finally, we discuss the ecological and methodological mechanisms that may obscure biodiversity–disease relationships and emphasize the need to adopt scale-explicit approaches, and to extend this scale-explicit framework to other host–pathogen systems.

## Materials and methods

### Biological model

Orthohantaviruses (enveloped, single-stranded, negative sense, RNA viruses belonging to the family *Hantaviridae*) constitute a particularly suitable biological model to characterise the effect of spatial scale on dilution/amplification effect. Hantaviruses are transmitted by various rodent species of the superfamily Muroidea in Europe, Asia, and the Americas [25], which are habitat generalists, geographically widespread, and locally highly abundant, making them well suited for large-scale comparative analyses. Moreover, despite several hantaviruses that tend to use several hosts [26], hantaviruses tend nevertheless to be “specialist”, *i.e.*, associated with only one given small-mammal species (especially rodents, shrews, moles, and bats) For example, Puumala virus (PUUV) in Europe is essentially restricted to the bank vole *Myodes glareolus*, Sin Nombre virus (SNV) in North America is primarily carried by the deer mouse *Peromyscus maniculatus*, and Andes virus in southern South America is associated with the long-tailed pygmy rice rat *Oligoryzomys longicaudatus*. By contrast, Seoul virus (SEOV), the dominant hantavirus genotype in our Asian dataset, infects several commensal *Rattus* species, and Tula virus (TULV) in Europe circulates across multiple *Microtus* voles, so that some hantaviruses fit a “multi-host” pattern. The richness term we model here is total rodent species richness in the cell, rather than the richness of competent reservoirs only, because for most non-reservoir species hantavirus competence has not been quantitatively assessed in the literature compiled by Milholland et al. [26,27]; we revisit this point in the Discussion [28]. This host adaptation pattern yields a large variability in host competence within rodent communities. In this context, the dilution effect has been observed several times for these hantaviruses, being especially evident when host species richness is high with an associated decrease in relative abundance of the most susceptible host species [29,30]. Moreover, hantaviruses represent a significant threat to human health. Transmission to humans occurs mainly via inhalation of aerosolized virus particles from infected rodent excreta or through direct contact with infected hosts, and can cause hemorrhagic fever with renal syndrome (mainly in Asia and Europe) and hantavirus pulmonary syndrome (mainly in the Americas) [31]. Thus, identifying the ecological determinants of the relationship between hantavirus prevalence and host species richness is crucial for understanding and preventing zoonotic disease risk in human populations.

### Dataset

The dataset used in this study is described by Milholland *et al. [*26,27*]*. In brief, this dataset comes from an extensive survey of the literature between 1971–2015 and comprises a global compilation of wild rodent species and their documented associations with hantaviruses. For each study, the dataset includes the number of individuals testing positive or negative for specific hantavirus species based on direct detection of viral genetic material (RNA). Accordingly, only host-virus associations confirmed by RT-PCR were retained. Although this molecular approach likely underestimates total host exposure compared to serology-based studies, it ensures a high specificity of host-virus associations by excluding serologic records in which specific hantavirus genotypes cannot be reliably identified. Therefore, data for each locality of the field study extracted involves sampling date, rodent species sampled, number of each hantavirus genotype identified within each rodent species, and total sample size for each rodent species. In total, the dataset contains exploitable data for three regions (North America, Asia, and Europe), encompassing 154 rodent species, 17,893 rodent individuals screened by RT-PCR (1,575 positives), and 19 hantavirus genotypes. A region-by-region breakdown of sampling effort, host species (including region-endemic species that tested positive), dominant hantavirus genotypes and main reservoir hosts is provided in S1 and S2 Tables.

### Statistical aggregation

**Fig 1:**
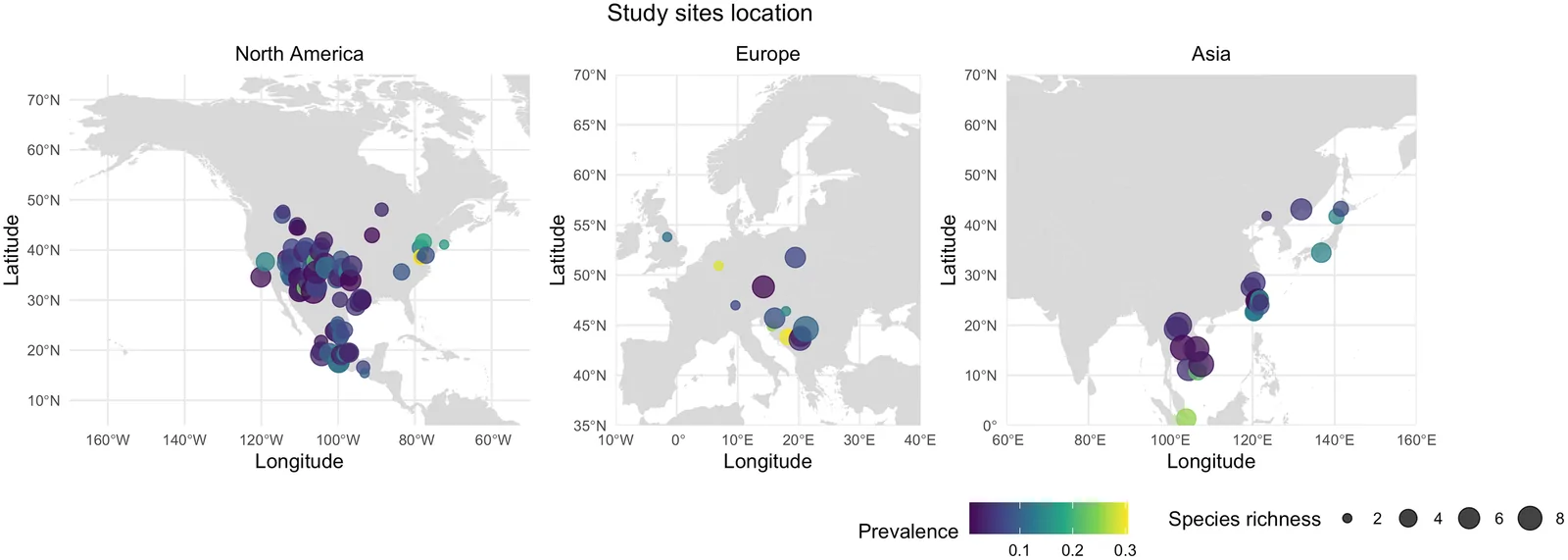
Spatial distribution of the sampling sites for Asia, Europe, and North America. Points are individual sampling localities from the source dataset [26,27]; no spatial aggregation is applied here. The size of the circles increases with the rodent species richness measured and the color represents the prevalence observed. Basemap: Natural Earth (public domain, https://www.naturalearthdata.com/), accessed via the rnaturalearth R package.

**Fig 2:**
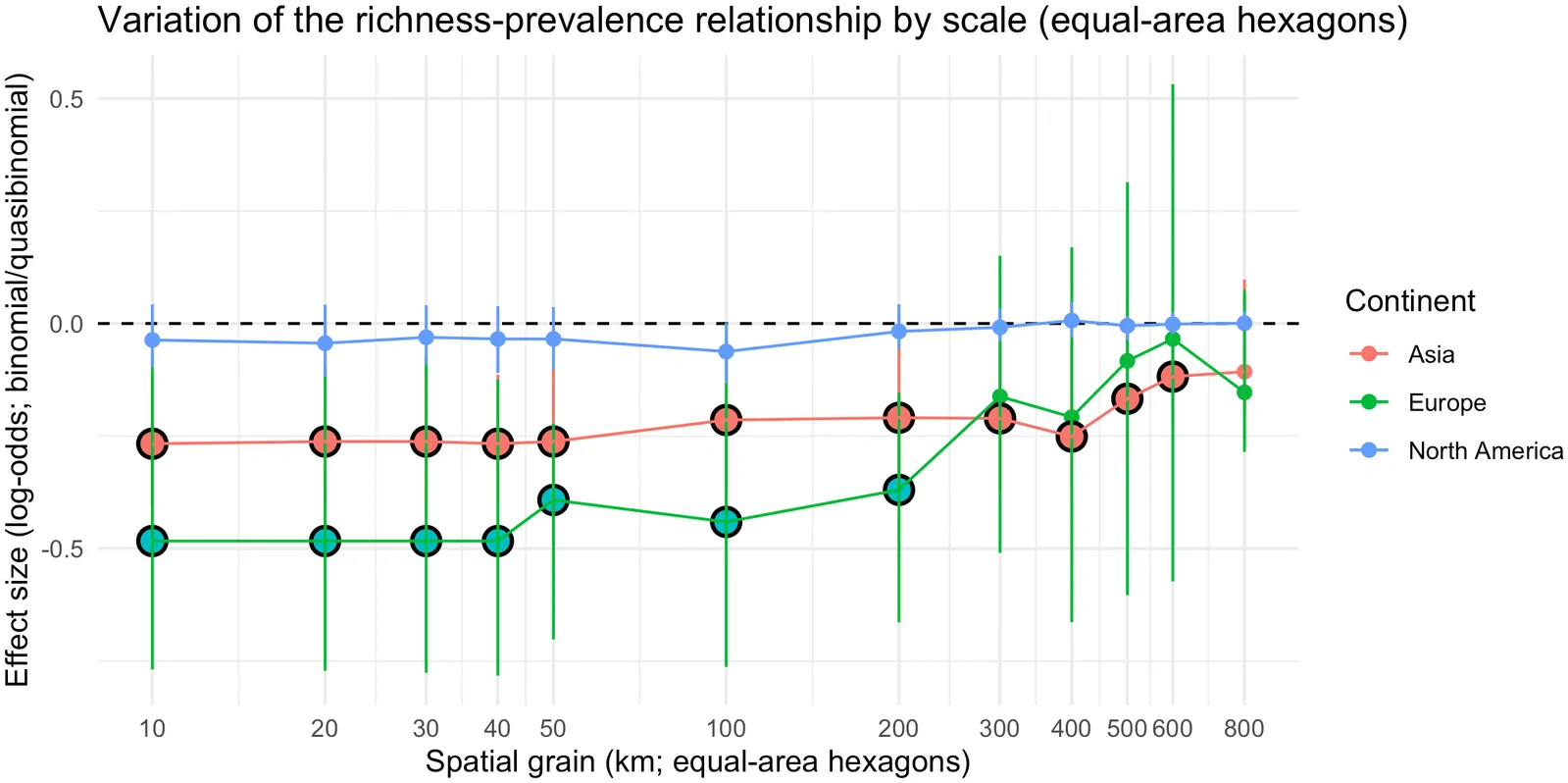
Relationship between dilution effect magnitude and the size of the spatial scale considered. Highlighted dots represent where the slope is significantly different from zero.

Spatial aggregation was performed using an equal-area hexagonal grid to ensure comparability of spatial units across latitudes. This avoids geometric distortions inherent to latitude–longitude grids defined in degrees, in which cell area varies with latitude, and provides a consistent definition of spatial scale. Hexagons were generated separately for each region in a Lambert Azimuthal Equal-Area (LAEA) projection centered on the mean longitude and latitude of that region’s sampling sites, in order to preserve cell area while minimising local distortion. Twelve grain sizes were evaluated, defined by the hexagon “diameter” (the distance between opposite vertices): 10, 20, 30, 40, 50, 100, 200, 300, 400, 500, 600 and 800 km. These grains span roughly two orders of magnitude in area, from sub-landscape (≈10 km) to sub-continental (≈800 km), and were chosen because they bracket the spatial extents at which biotic interactions among rodent hosts and large-scale climatic constraints respectively dominate community assembly. For each region and each grain, only cells containing at least two rodent species and a non-zero sampling effort (N > 0) were retained in the analysis. Fig 1 displays the raw, unaggregated sampling sites (i.e., point coordinates from the source dataset, with no spatial pooling); aggregation into hexagonal cells was applied only to the statistical analysis underlying Fig 2.

Spatial grain was systematically increased by varying hexagonal cell size, thereby pooling progressively larger areas while maintaining constant cell area within each resolution. This approach allows spatial scale to be interpreted unambiguously in terms of area rather than angular distance.

### Statistical analyses

Infection prevalence was modeled using a binomial generalized linear modeling framework in which the number of infected individuals and the total number sampled within each spatial unit were explicitly represented. This approach accounts for the bounded nature of prevalence data and for heterogeneity in sampling effort among spatial units.

To accommodate potential overdispersion arising from aggregation across multiple host species and study sites, a quasi-binomial variance was specified whenever the residual deviance dispersion exceeded 1.5, so that standard errors and p-values are inflated to account for extra-binomial variability. Within each region and at each spatial grain, the relationship between hantavirus prevalence and rodent species richness was modelled by:


$$\begin{array}{c} Y_{i}\;\sim \text{ Binomial}(N_{i},\;p_{i}),\text{ logit}(p_{i})=\beta_{\text{0}}+\beta_{\text{1}}\times {\text{richness}}_{\text{i}} \end{array}$$

where Yi is the number of hantavirus-positive rodents in cell i, Ni is the total number of rodents screened in that cell, richnessi is the observed number of rodent species in the cell, β0 is the intercept, and β1 is the slope of interest, i.e., the log-odds change in prevalence per additional rodent species. A negative β1 indicates a dilution effect, a positive β1 an amplification effect. Models were fitted independently for each region (Asia, Europe, North America) at each spatial grain, with no pooling across regions, so that the estimated coefficient and its overdispersion-adjusted standard error reflect within-region variation only. Within each region, P-values across the twelve spatial grains were adjusted for multiple comparisons using the Benjamini-Hochberg procedure. In a complementary analysis (S4 Table), we also fitted a single pooled binomial generalised linear mixed model at each grain, with continent and cell as random intercepts, to confirm that the within-region patterns were not driven by between-region heterogeneity in baseline prevalence.

Uncertainty around effect-size estimates was quantified using non-parametric bootstrap resampling (1,000 iterations) conducted independently within each region and spatial resolution. For each bootstrap replicate, spatial units were resampled with replacement and the richness coefficient was re-estimated. Percentile and bias-corrected accelerated (BCa) confidence intervals were derived from the bootstrap distributions.

## Results

The studies included in the analysis are well distributed across the three regions considered, namely North America, Europe, and Asia. Sampling locations cover the full spatial extent of each continent, ensuring a balanced representation of the study areas and reducing potential biases associated with uneven geographic coverage (Fig 1). The North American dataset is the largest (91 unique sampling sites, 110 rodent species tested, 10,396 individuals screened, overall RT-PCR positivity 7.7%) and is dominated by Sin Nombre virus (464 of 796 positive detections) and by its main reservoir the deer mouse Peromyscus maniculatus (341 of 796 positives). The Asian dataset comprises 28 sites, 36 species and 6,461 individuals tested (overall positivity 9.2%) and is dominated by Seoul virus (498 of 593 positives), carried mostly by Rattus norvegicus. The European dataset is smaller (13 sites, 20 species, 1,036 individuals) and shows the highest overall positivity (18.0%), with Puumala virus and Tula virus contributing roughly equally and reservoir hosts dominated by Myodes glareolus and Apodemus flavicollis. A region-by-region descriptive breakdown, including the number of region-endemic rodent species that tested positive at least once (16 in Asia, 7 in Europe, 35 in North America), is provided in S1 and S2 Tables.

In Asia and Europe, the estimated effect of rodent species richness on hantavirus prevalence was negative across all spatial grains and statistically significant at fine resolutions, with the largest absolute effect sizes observed in Europe at grains ≤ 50 km (β = −0.48, SE = 0.14, BCa 95% CI [−0.78; −0.07], BH-adjusted P = 0.013 at 10–40 km) and in Asia at grains ≤ 50 km (β ≈ −0.26 to −0.29, BH-adjusted P ≤ 0.005). As spatial grain increased, the magnitude of the effect progressively decreased and the BCa confidence intervals widened to overlap zero: in Europe, the dilution effect lost statistical significance from 300 km onwards (β = −0.16, BH-adjusted P = 0.24); in Asia, the effect remained significant up to 600 km (β = −0.12, BH-adjusted P = 0.021) and only became non-significant at 800 km (β = −0.11, BH-adjusted P = 0.062). In North America, the estimated effect was also predominantly negative at most grains but was systematically weaker in absolute value (|β| ≤ 0.07 across all scales) and never reached statistical significance after Benjamini-Hochberg correction (smallest BH-adjusted P = 0.13 at 100 km).

The full set of effect sizes, standard errors, BCa confidence intervals, raw and BH-adjusted p-values, and dispersion statistics for each region × grain combination is reported in S3 Table and visualised in Fig 2. The complementary pooled binomial GLMM with continent and cell as random intercepts (S4 Table) confirms this scale-dependent pattern across the three regions combined: the pooled richness coefficient is strongly negative and highly significant at fine grains (β ≈ −0.10, BH-adjusted P < 0.0001 at 10–100 km), progressively attenuates between 200 and 300 km, and is no longer distinguishable from zero (or marginally positive) from 400 km onwards.

## Discussion

In this study, we evaluated how spatial scale influences the detection of the relationship between host species richness and hantavirus prevalence. Across all regions considered, the relationship between rodent species richness and hantavirus infection prevalence is predominantly negative, consistent with a dilution effect, while positive relationships are rare and never statistically significant. However, increasing spatial aggregation leads to a systematic decrease in the magnitude of the dilution effect, which can become statistically undetectable at coarse spatial scales. Thus, although the direction of the relationship remains negative, its probability of statistical detection decreases as spatial scale increases. Previous work on other disease systems has more generally established that biodiversity-disease relationships are sensitive to spatial scale, even when the precise pattern (progressive weakening of fine-scale dilution at coarser grains) is not always the one reported here [32,33].

These results can be explained by the relative influence of biotic and abiotic processes on host community structure and pathogen transmission. Because the dilution effect is primarily driven by biotic interactions among host species, including contact structure, relative host abundances, and community composition [20,34], it is expected to be strongest at fine spatial scales, where such interactions directly shape transmission dynamics. This interpretation is consistent with studies showing that host-host interactions and network structure play a dominant role in regulating pathogen transmission, whereas broader-scale patterns increasingly reflect the influence of abiotic and landscape-level constraints [35,36]. In contrast, theory predicts that at broader spatial scales, abiotic drivers such as climate increasingly constrain species distributions and abundances (*e.g.*, McGill 2010). As a consequence, spatial aggregation tends to pool communities structured by similar environmental conditions, leading to more homogeneous assemblages than those observed at finer scales. This homogenization can weaken and mask the detectable relationship between host species richness and pathogen prevalence at large spatial scales, as observed in our analysis.

Our findings align with previous work while providing additional resolution on the role of spatial scale in biodiversity-disease relationships. For example, Magnusson et al. (2020) reported a generally negative association between host species richness and pathogen prevalence across multiple spatial extents and geographic regions [23]. Our analysis confirms this overall pattern, but further reveals how spatial aggregation progressively attenuates the magnitude and detectability of the dilution effect. By varying spatial scale in a continuous manner rather than using broad categorical units (*e.g.*, country or continent), we demonstrate that increasing aggregation progressively attenuates the dilution effect, eventually reducing its statistical detectability. This refinement highlights how scale-dependent processes can be obscured when spatial resolution is treated too coarsely.

Our scale-explicit re-analysis of a global compiled dataset provides a quantitative assessment of how spatial scale alters the relationship between biodiversity and pathogen prevalence, using hantaviruses as a focal system. In hantavirus systems, previous studies emphasize the role of host community composition and habitat structure in modulating transmission dynamics, while also highlighting the modulatory influence of environmental conditions and host population dynamics [37–39]. Although our findings are likely applicable to other directly transmitted pathogens, it remains unclear whether similar scale-dependent responses would occur for pathogens with different transmission modes, such as vector-borne diseases, for which additional ecological and epidemiological processes may influence the emergence or attenuation of dilution effects. Focusing on a single pathogen group allows for robust and interpretable comparisons of prevalence across studies, whereas integrating multiple pathogens introduces additional sources of heterogeneity that a complicate inference. Nevertheless, applying this scale-explicit framework to other pathogen systems will be necessary to build a more comprehensive understanding of how spatial scale shapes the detection of biodiversity–disease relationships.

Importantly, the scale-dependent attenuation of the dilution effect observed here is robust to alternative statistical and spatial representations. By explicitly modeling infection counts using a binomial framework and aggregating data within equal-area spatial units, we demonstrate that the weakening of the richness–prevalence relationship with increasing spatial grain is not an artifact of prevalence bounds, uneven sampling effort, or geometric distortions. Instead, it reflects a genuine scale dependence in the detectability of biodiversity–disease relationships, consistent with shifts in the dominant ecological processes governing host community structure and pathogen transmission across spatial scales.

The absence of a significant dilution effect in North America deserves particular attention. In our dataset, North American hantavirus circulation is overwhelmingly dominated by Sin Nombre virus, with the deer mouse *Peromyscus maniculatus* contributing 43% of all positive detections in the region. When a single, abundant and widely distributed competent host carries most of the transmission, the addition of further rodent species in a cell has limited potential to dilute prevalence, because the dominant host typically remains numerically and epidemiologically dominant regardless of community richness. The strong asymmetry of host competence in the SNV-*P. maniculatus* system therefore likely places a structural ceiling on the detectable dilution signal at the continental level. We also explored whether pooling temperate United States with tropical Mexico (two ecoregions with markedly different climatic and faunal contexts) might contribute to the weak North American signal, through a latitudinal sensitivity analysis splitting the North American records at the Tropic of Cancer (~23.43°N) (S1 Fig and S5 Table). The point estimates suggest an apparent convergence of effect sizes at fine grains in the two subsets (β ≈ −0.05 at 10–100 km in both the temperate and the tropical subset). However, the BCa 95% confidence intervals on these subset-specific estimates overlap zero at every grain, and the tropical subset is supported by only 5–14 spatial cells, so this analysis cannot be considered statistically robust. It is best read as a tentative indication that a latitudinal aggregation artefact may contribute, alongside the dominance of *P. maniculatus*/SNV, to the weak continent-level signal, but a confirmatory test of this hypothesis will require additional sampling from southern Mexico and Central America.

Two important methodological caveats should be made explicit. First, the source dataset compiled by Milholland et al. does not provide systematic, RT-PCR-confirmed records for South America, and South American hantavirus systems (e.g., Andes virus and other genotypes circulating in diverse sigmodontine rodent communities) are therefore not represented in our analysis. Because southern South America hosts both a particularly diverse sigmodontine fauna and ecologically distinct hantavirus lineages, extending the scale-explicit framework presented here to South American data is a clear priority. Second, our richness predictor is the total number of rodent species observed in a cell, not the richness of competent reservoirs nor a measure of community composition or competition. A dilution effect may operate primarily through the replacement of highly competent hosts by less competent ones, rather than through species number per se. With the current dataset, host-specific competence is rarely quantified outside of a few well-studied reservoirs (*P. maniculatus* for SNV, *M. glareolus* for PUUV, *R. norvegicus* for SEOV), so a competence-weighted richness metric could not be reliably constructed. The patterns reported here are therefore best interpreted as describing how the empirical relationship between total host species richness and prevalence behaves across scales, and not as a mechanistic test of competence-mediated dilution. Relatedly, spatial aggregation at coarser grains progressively mixes cells where different dominant hantavirus genotypes circulate (e.g., SEOV-dominated cells pooled with PUUV-dominated cells in Asia, or SNV-dominated cells pooled with Bayou and Black Creek Canal cells in North America). This virus-mixing is itself a mechanism by which large-scale aggregation can attenuate the apparent richness-prevalence relationship, in addition to the abiotic-driver mechanism discussed above.

A further mechanism that contributes to the scale-dependent attenuation we observe is the species-area relationship. Because the number of species sampled within a cell increases with cell area, the variance in richness at coarse grains is compressed: most large cells eventually contain similarly high richness, which mechanically reduces the slope of the prevalence-richness relationship even in the absence of any change in the underlying biotic processes. We checked this directly by regressing log(mean species richness) on log(spatial grain) within each region (S6 Table). A significant positive species-area scaling is found in all three regions (Asia: z = 0.054, P < 0.001, R² = 0.81; Europe: z = 0.078, P < 0.001, R² = 0.70; North America: z = 0.178, P < 0.0001, R² = 0.85), consistent with classical biogeographic expectations. The slope is more than three times larger in North America than in Asia, which is consistent with the much wider latitudinal extent of the North American sampling frame and reinforces the interpretation that part of the attenuation observed there reflects mechanical species accumulation at coarse grains. The weakening of the dilution effect at coarser grains should therefore be interpreted as the joint outcome of (i) homogenisation of communities pooled across environmental gradients, (ii) virus-mixing across cells dominated by different reservoir hosts, and (iii) a species-area-driven compression of the richness gradient itself. None of these mechanisms invalidates the dilution effect at fine scales, but they jointly explain why coarse-grain analyses can fail to detect it.

From an applied perspective, a scale-aware public health framework for hantavirus prevention would mean targeting rodent surveillance and habitat-management interventions at the landscape grain at which the dilution effect is empirically detectable (here, of the order of tens of kilometres), rather than at the country or continental level at which most existing surveillance is organised. Concretely, this could take the form of municipality-level or watershed-level rodent monitoring programmes that record both community composition and hantavirus prevalence, paired with land-use interventions (preserving habitat heterogeneity in peri-urban zones, limiting the conversion of mature habitats to monoculture or simplified agro-ecosystems) that maintain non-reservoir species in the local rodent community. A coarser, national-level metric of “rodent biodiversity” would not capture the variation that matters for transmission risk and could mask actionable signals.

Recent critiques have questioned the generality and falsifiability of the dilution effect, emphasizing that biodiversity-disease relationships are inherently context-dependent and highly sensitive to spatial scale and analytical resolution [40,41]. Our results are consistent with this perspective, as the attenuation of dilution effects at broader spatial scales likely reflects shifts in the dominant ecological processes shaping transmission, rather than the absence of biodiversity-mediated processes influencing transmission.

In conclusion, our study shows that the direction of the biodiversity–disease relationship, as reflected by the presence of a dilution effect, is not contingent on the spatial scale considered. This finding broadens the scope for investigating dilution effects across a wider range of biological systems than previously explored, thereby facilitating assessments of their generality. In addition, our results indicate that higher host biodiversity is associated with reduced prevalence of a directly transmitted zoonotic pathogen. These results highlight the need to match conservation and public health interventions to the spatial scales at which ecological processes driving disease transmission operate. In this context, maintaining host biodiversity may contribute to reducing the prevalence of directly transmitted zoonotic pathogens and should be considered as part of integrated, conservation strategy, and scale-aware public health policy frameworks.

## Supporting information

S1 Table Region-by-region summary of the cleaned dataset. (DOCX)

S2 Table Dominant hantavirus genotypes and main reservoir hosts per region. (DOCX)

S3 Table Region-stratified effect of rodent species richness on hantavirus prevalence at each spatial grain. (DOCX)

S4 Table Pooled binomial GLMM with continent and cell as random intercepts. (DOCX)

S5 Table Latitudinal sensitivity analysis: temperate vs tropical North America. (DOCX)

S6 Table Species-area relationship: scaling of mean species richness with spatial grain. (DOCX)

S1 Fig Latitudinal sensitivity analysis: temperate vs tropical North America. (DOCX)
