# Supplementary material for: Increasing spatial scale of analysis weakens and can mask the dilution effect for hantaviruses
Source: PLOS Glob Public Health. 2026 Sep 8;6(9):e0007045. doi: 10.1371/journal.pgph.0007045 (PMC13552722; doi:10.1371/journal.pgph.0007045)
Supplement: Supplementary file 1 [file pgph.0007045.s001.docx]

**Table S1. Region-by-region summary of the cleaned dataset**

Number of unique sampling sites, rodent species tested, rodent species that returned at least one positive RT-PCR result, region-endemic species (species recorded in this region only), region-endemic species with at least one positive detection, hantavirus genotypes identified, total individuals screened, total positive detections, and overall RT-PCR positivity for each region.

| Region | N sampling sites | N rodent species tested | N rodent species positive (at least 1) | N region-endemic species | N region-endemic species positive | N hantavirus genotypes detected | Total rodents tested | Total positive | Overall prevalence (%) |
| --- | --- | --- | --- | --- | --- | --- | --- | --- | --- |
| Asia | 28 | 36 | 20 | 29 | 16 | 6 | 6461 | 593 | 9.18 |
| Europe | 13 | 20 | 10 | 14 | 7 | 5 | 1036 | 186 | 17.95 |
| North America | 91 | 110 | 38 | 103 | 35 | 10 | 10396 | 796 | 7.66 |
