# Supplementary material for: Increasing spatial scale of analysis weakens and can mask the dilution effect for hantaviruses
Source: PLOS Glob Public Health. 2026 Sep 8;6(9):e0007045. doi: 10.1371/journal.pgph.0007045 (PMC13552722; doi:10.1371/journal.pgph.0007045)
Supplement: Supplementary file 2 [file pgph.0007045.s002.docx]

**Table S2. Dominant hantavirus genotypes and main reservoir hosts per region**

For each region, the three hantavirus genotypes with the largest number of RT-PCR positive detections (with n positives in parentheses), and the three rodent species accounting for the largest number of positives. Species names are given in upper case as they appear in the source dataset of Milholland et al. (2018, 2019); the corresponding genus is implicit from the dataset.

| Region | Dominant hantaviruses (n positives) | Main reservoir hosts by positives (n) |
| --- | --- | --- |
| Asia | SEOV (498), PUUV (23), THIV (11) | Norvegicus (435), Rattus (32), Rufocanus (24) |
| Europe | PUUV (23), TULV (23), DOBV (4) | Flavicollis (67), Glareolus (57), Arvalis (15) |
| North America | SNV (464), BAYV (97), OROV (29) | Maniculatus (341), Palustris (140), Boylii (72) |
