# Supplementary material for: Increasing spatial scale of analysis weakens and can mask the dilution effect for hantaviruses
Source: PLOS Glob Public Health. 2026 Sep 8;6(9):e0007045. doi: 10.1371/journal.pgph.0007045 (PMC13552722; doi:10.1371/journal.pgph.0007045)
Supplement: Supplementary file 3 [file pgph.0007045.s003.docx]

**Table S3. Region-stratified effect of rodent species richness on hantavirus prevalence at each spatial grain**

Per-region binomial / quasi-binomial GLM estimates underlying Figure 2 of the main text. β is the log-odds change in prevalence per additional rodent species in the cell. Standard errors and p-values are overdispersion-adjusted (quasi-binomial) where the residual deviance dispersion exceeds 1.5. BCa 95% CIs are derived from 1,000 non-parametric bootstrap resamples of cells. P-values are adjusted within each region across the twelve spatial grains by the Benjamini-Hochberg procedure.

| continent | Spatial grain (km) | Number of cells | Mean prevalence | Mean species richness | β (log-odds) | SE | P-value | P-value (BH) | Significant (BH < 0.05) | BCa 95% CI lower | BCa 95% CI upper | Dispersion | Model |
| --- | --- | --- | --- | --- | --- | --- | --- | --- | --- | --- | --- | --- | --- |
| Asia | 10 | 27 | 0.104 | 4.96 | -0.267 | 0.071 | 9.00e-04 | 0.0039 | Yes | -0.404 | -0.126 | 9.09 | quasibinomial |
| Asia | 20 | 25 | 0.096 | 5.16 | -0.262 | 0.075 | 0.0019 | 0.0045 | Yes | -0.398 | -0.091 | 10.15 | quasibinomial |
| Asia | 30 | 25 | 0.096 | 5.16 | -0.262 | 0.075 | 0.0019 | 0.0045 | Yes | -0.413 | -0.096 | 10.15 | quasibinomial |
| Asia | 40 | 27 | 0.104 | 4.96 | -0.267 | 0.071 | 9.00e-04 | 0.0039 | Yes | -0.398 | -0.114 | 9.1 | quasibinomial |
| Asia | 50 | 24 | 0.091 | 5.29 | -0.262 | 0.076 | 0.0023 | 0.0047 | Yes | -0.434 | -0.101 | 10.6 | quasibinomial |
| Asia | 100 | 24 | 0.098 | 5.25 | -0.214 | 0.075 | 0.0092 | 0.0137 | Yes | -0.374 | -0.036 | 11.34 | quasibinomial |
| Asia | 200 | 17 | 0.096 | 5.65 | -0.209 | 0.061 | 0.0037 | 0.0064 | Yes | -0.371 | -0.051 | 8.88 | quasibinomial |
| Asia | 300 | 16 | 0.089 | 5.94 | -0.211 | 0.073 | 0.012 | 0.016 | Yes | -0.372 | -0.045 | 13.56 | quasibinomial |
| Asia | 400 | 20 | 0.099 | 5.45 | -0.251 | 0.064 | 0.001 | 0.0039 | Yes | -0.366 | -0.046 | 7.1 | quasibinomial |
| Asia | 500 | 14 | 0.099 | 5.93 | -0.167 | 0.062 | 0.0189 | 0.0207 | Yes | -0.291 | 0.062 | 10.61 | quasibinomial |
| Asia | 600 | 13 | 0.101 | 6.23 | -0.118 | 0.042 | 0.0175 | 0.0207 | Yes | -0.212 | 0.045 | 12.33 | quasibinomial |
| Asia | 800 | 11 | 0.1 | 6.45 | -0.107 | 0.05 | 0.0619 | 0.0619 | No | -0.202 | 0.098 | 13.52 | quasibinomial |
| Europe | 10 | 13 | 0.214 | 4.85 | -0.483 | 0.14 | 0.0055 | 0.0131 | Yes | -0.769 | -0.066 | 6.14 | quasibinomial |
| Europe | 20 | 13 | 0.214 | 4.85 | -0.483 | 0.14 | 0.0055 | 0.0131 | Yes | -0.771 | -0.111 | 6.14 | quasibinomial |
| Europe | 30 | 13 | 0.214 | 4.85 | -0.483 | 0.14 | 0.0055 | 0.0131 | Yes | -0.776 | -0.075 | 6.14 | quasibinomial |
| Europe | 40 | 13 | 0.214 | 4.85 | -0.483 | 0.14 | 0.0055 | 0.0131 | Yes | -0.782 | -0.124 | 6.14 | quasibinomial |
| Europe | 50 | 12 | 0.226 | 5 | -0.392 | 0.096 | 0.0023 | 0.0131 | Yes | -0.702 | -0.223 | 5.68 | quasibinomial |
| Europe | 100 | 12 | 0.194 | 5.08 | -0.441 | 0.16 | 0.0202 | 0.0346 | Yes | -0.763 | -0.004 | 8.95 | quasibinomial |
| Europe | 200 | 11 | 0.206 | 5.27 | -0.37 | 0.115 | 0.0104 | 0.0208 | Yes | -0.664 | -0.153 | 8.14 | quasibinomial |
| Europe | 300 | 10 | 0.205 | 5.6 | -0.162 | 0.116 | 0.199 | 0.2389 | No | -0.51 | 0.151 | 11.46 | quasibinomial |
| Europe | 400 | 9 | 0.19 | 5.44 | -0.208 | 0.137 | 0.1727 | 0.2303 | No | -0.664 | 0.17 | 17.05 | quasibinomial |
| Europe | 500 | 8 | 0.194 | 5.75 | -0.083 | 0.078 | 0.3276 | 0.3574 | No | -0.604 | 0.314 | 15.68 | quasibinomial |
| Europe | 600 | 7 | 0.194 | 6.71 | -0.034 | 0.139 | 0.8149 | 0.8149 | No | -0.572 | 0.546 | 24.11 | quasibinomial |
| Europe | 800 | 5 | 0.244 | 7.4 | -0.153 | 0.058 | 0.0782 | 0.1174 | No | -0.285 | 0.066 | 8.78 | quasibinomial |
| North America | 10 | 85 | 0.099 | 5.71 | -0.037 | 0.024 | 0.1341 | 0.3822 | No | -0.097 | 0.043 | 3.75 | quasibinomial |
| North America | 20 | 83 | 0.098 | 5.82 | -0.044 | 0.025 | 0.0835 | 0.3822 | No | -0.117 | 0.042 | 3.72 | quasibinomial |
| North America | 30 | 79 | 0.097 | 5.86 | -0.031 | 0.024 | 0.21 | 0.4201 | No | -0.09 | 0.041 | 4.01 | quasibinomial |
| North America | 40 | 80 | 0.095 | 5.89 | -0.034 | 0.024 | 0.1593 | 0.3822 | No | -0.11 | 0.039 | 3.8 | quasibinomial |
| North America | 50 | 76 | 0.095 | 5.96 | -0.034 | 0.024 | 0.1582 | 0.3822 | No | -0.102 | 0.036 | 3.96 | quasibinomial |
| North America | 100 | 73 | 0.098 | 6.15 | -0.062 | 0.024 | 0.0111 | 0.1337 | No | -0.133 | 0.002 | 3.7 | quasibinomial |
| North America | 200 | 58 | 0.098 | 7.03 | -0.018 | 0.017 | 0.3166 | 0.5427 | No | -0.056 | 0.043 | 4.13 | quasibinomial |
| North America | 300 | 42 | 0.092 | 8.43 | -0.008 | 0.014 | 0.5484 | 0.7873 | No | -0.04 | 0.032 | 3.88 | quasibinomial |
| North America | 400 | 36 | 0.089 | 9.06 | 0.007 | 0.013 | 0.6024 | 0.7873 | No | -0.03 | 0.048 | 4.43 | quasibinomial |
| North America | 500 | 30 | 0.09 | 9.9 | -0.005 | 0.011 | 0.6561 | 0.7873 | No | -0.037 | 0.016 | 4.07 | quasibinomial |
| North America | 600 | 24 | 0.09 | 10.88 | -0.001 | 0.007 | 0.8429 | 0.9143 | No | -0.024 | 0.025 | 5.18 | quasibinomial |
| North America | 800 | 18 | 0.107 | 12.72 | 0.001 | 0.007 | 0.9143 | 0.9143 | No | -0.015 | 0.027 | 4.99 | quasibinomial |
