# Supplementary material for: Increasing spatial scale of analysis weakens and can mask the dilution effect for hantaviruses
Source: PLOS Glob Public Health. 2026 Sep 8;6(9):e0007045. doi: 10.1371/journal.pgph.0007045 (PMC13552722; doi:10.1371/journal.pgph.0007045)
Supplement: Supplementary file 4 [file pgph.0007045.s004.docx]

**Table S4. Pooled binomial GLMM with continent and cell as random intercepts**

Complementary analysis pooling all three regions at each spatial grain. The implemented model is cbind(n_positive, n_tested-n_positive) ~ species_count + (1|continent) + (1|cell_id), fitted with lme4::glmer (binomial family) in the R pipeline (HantaV6.R). The numbers reported here were computed in Python with continent as a fixed effect, because with only three continent levels the random-intercept variance is very poorly identified; the R GLMM converges to essentially the same fixed-effect coefficient. β is the slope of species_count, SE its standard error (overdispersion-corrected when dispersion > 1.5), and BH-adjusted p-values are reported across the twelve spatial grains.

| scale_km | n_cells_pooled | beta | SE | CI95_lo | CI95_hi | p_value | dispersion | model | p_adj_BH | significant_BH |
| --- | --- | --- | --- | --- | --- | --- | --- | --- | --- | --- |
| 10 | 118 | -0.0987 | 0.0029 | -0.1044 | -0.093 | 0 | 7.0524 | quasibinomial | 0 | Yes |
| 20 | 119 | -0.104 | 0.0029 | -0.1097 | -0.0983 | 0 | 6.9209 | quasibinomial | 0 | Yes |
| 30 | 114 | -0.0985 | 0.0029 | -0.1042 | -0.0929 | 0 | 7.1722 | quasibinomial | 0 | Yes |
| 40 | 115 | -0.1011 | 0.0028 | -0.1066 | -0.0955 | 0 | 7.0806 | quasibinomial | 0 | Yes |
| 50 | 108 | -0.0959 | 0.0027 | -0.1012 | -0.0905 | 0 | 7.2476 | quasibinomial | 0 | Yes |
| 100 | 87 | -0.1041 | 0.0026 | -0.1091 | -0.0991 | 0 | 7.9354 | quasibinomial | 0 | Yes |
| 200 | 66 | -0.0286 | 0.0018 | -0.0321 | -0.0251 | 0 | 8.7864 | quasibinomial | 0 | Yes |
| 300 | 49 | -0.0189 | 0.0013 | -0.0215 | -0.0164 | 0 | 11.4355 | quasibinomial | 0 | Yes |
| 400 | 37 | -7.00e-04 | 9.00e-04 | -0.0024 | 0.001 | 0.4005 | 9.1765 | quasibinomial | 0.4005 | No |
| 500 | 34 | 0.0058 | 7.00e-04 | 0.0045 | 0.0072 | 0 | 10.1267 | quasibinomial | 0 | Yes |
| 600 | 29 | 0.0081 | 6.00e-04 | 0.007 | 0.0092 | 0 | 11.3019 | quasibinomial | 0 | Yes |
| 800 | 23 | 0.0032 | 5.00e-04 | 0.0022 | 0.0042 | 0 | 14.3931 | quasibinomial | 0 | Yes |
