# Supplementary material for: Increasing spatial scale of analysis weakens and can mask the dilution effect for hantaviruses
Source: PLOS Glob Public Health. 2026 Sep 8;6(9):e0007045. doi: 10.1371/journal.pgph.0007045 (PMC13552722; doi:10.1371/journal.pgph.0007045)
Supplement: Supplementary file 5 [file pgph.0007045.s005.docx]

**Table S5. Latitudinal sensitivity analysis: temperate vs tropical North America**

North American records were split at the Tropic of Cancer (~23.43°N) into a temperate subset (USA + northern Mexico, n = 478 records, 86 rodent species, 73 sampling sites) and a tropical subset (southern Mexico, n = 86 records, 35 species, 18 sampling sites). The same binomial / quasi-binomial GLM as in Table S3 was then refitted within each subset at each spatial grain. Reading note: at fine grains (10-100 km) the point estimates of the richness effect are very similar in the two subsets (β ≈ -0.05) and are flagged as statistically significant by the quasi-binomial BH-adjusted test. However, the BCa 95% confidence intervals (1,000 bootstrap resamples of cells) overlap zero at every grain in both subsets. The tropical subset is also supported by only 5 to 14 spatial cells, which strongly limits its statistical power and makes the apparent flip towards a positive coefficient at 200-500 km (β ≈ +0.04 to +0.10) very sensitive to the inclusion or exclusion of a few cells. This analysis is therefore best read as descriptive: it suggests that part of the weak continent-level North American signal could reflect a latitudinal aggregation artefact, but the available data do not allow this hypothesis to be confirmed.

| zone | scale_km | n_cells | beta | SE | p_value | BCa_lo | BCa_hi | p_adj_BH | significant_BH | significant_BCa |
| --- | --- | --- | --- | --- | --- | --- | --- | --- | --- | --- |
| NA_temperate | 10 | 69 | -0.0456 | 0.0037 | 0 | -0.1393 | 0.0207 | 0 | Yes | No |
| NA_temperate | 20 | 64 | -0.0458 | 0.0036 | 0 | -0.1432 | 0.017 | 0 | Yes | No |
| NA_temperate | 30 | 61 | -0.0435 | 0.0036 | 0 | -0.1348 | 0.018 | 0 | Yes | No |
| NA_temperate | 40 | 60 | -0.0443 | 0.0036 | 0 | -0.1371 | 0.019 | 0 | Yes | No |
| NA_temperate | 50 | 59 | -0.0553 | 0.0036 | 0 | -0.1179 | 0.0436 | 0 | Yes | No |
| NA_temperate | 100 | 50 | -0.0556 | 0.003 | 0 | -0.1199 | 0.0018 | 0 | Yes | No |
| NA_temperate | 200 | 33 | 0.0023 | 0.0013 | 0.08 | -0.0417 | 0.057 | 0.096 | No | No |
| NA_temperate | 300 | 23 | 0.0018 | 9.00e-04 | 0.0429 | -0.0369 | 0.0218 | 0.0573 | No | No |
| NA_temperate | 400 | 17 | 7.00e-04 | 7.00e-04 | 0.2936 | -0.0272 | 0.0194 | 0.3203 | No | No |
| NA_temperate | 500 | 14 | 0.0032 | 8.00e-04 | 1.00e-04 | -0.0134 | 0.0468 | 1.00e-04 | Yes | No |
| NA_temperate | 600 | 12 | 0.005 | 8.00e-04 | 0 | -0.0321 | 0.022 | 0 | Yes | No |
| NA_temperate | 800 | 9 | 2.00e-04 | 4.00e-04 | 0.6349 | -0.0189 | 0.0582 | 0.6349 | No | No |
| NA_tropical | 10 | 14 | -0.0545 | 0.0071 | 0 | -0.1861 | 0.0901 | 0 | Yes | No |
| NA_tropical | 20 | 13 | -0.0455 | 0.007 | 0 | -0.1665 | 0.1032 | 0 | Yes | No |
| NA_tropical | 30 | 14 | -0.0545 | 0.0071 | 0 | -0.192 | 0.0935 | 0 | Yes | No |
| NA_tropical | 40 | 13 | -0.0455 | 0.007 | 0 | -0.1742 | 0.0997 | 0 | Yes | No |
| NA_tropical | 50 | 13 | -0.0455 | 0.007 | 0 | -0.1826 | 0.0942 | 0 | Yes | No |
| NA_tropical | 100 | 10 | -0.0514 | 0.0078 | 0 | -0.195 | 0.1224 | 0 | Yes | No |
| NA_tropical | 200 | 7 | 0.0411 | 0.0055 | 0 | -0.0946 | 0.249 | 0 | Yes | No |
| NA_tropical | 300 | 5 | 0.099 | 0.0093 | 0 | -0.0577 | 0.2461 | 0 | Yes | No |
| NA_tropical | 400 | 5 | 0.099 | 0.0093 | 0 | -0.0577 | 0.2462 | 0 | Yes | No |
| NA_tropical | 500 | 5 | 0.099 | 0.0093 | 0 | -0.0863 | 0.2457 | 0 | Yes | No |
| NA_tropical | 600 | 4 | — | — | — | — | — | — | No | No |
| NA_tropical | 800 | 4 | — | — | — | — | — | — | No | No |
