# Supplementary material for: Increasing spatial scale of analysis weakens and can mask the dilution effect for hantaviruses
Source: PLOS Glob Public Health. 2026 Sep 8;6(9):e0007045. doi: 10.1371/journal.pgph.0007045 (PMC13552722; doi:10.1371/journal.pgph.0007045)
Supplement: Supplementary file 6 [file pgph.0007045.s006.docx]

**Table S6. Species-area relationship: scaling of mean species richness with spatial grain**

Within each region, log(mean species richness per cell) was regressed on log(spatial grain in km) across the twelve grains analysed (10-800 km). SAR_slope_z is the slope on the log-log scale (the exponent z of the classical species-area relationship S = c·A^z, here approximated using cell diameter as a proxy for area). All three regions show a positive and statistically significant species-area scaling, indicating that part of the attenuation of the dilution effect at coarser grains reflects a mechanical accumulation of species with area rather than a change in the underlying ecological process.

| continent | n_grains | SAR_slope_z | SAR_SE | SAR_p_value | R_squared | intercept |
| --- | --- | --- | --- | --- | --- | --- |
| Asia | 12 | 0.0539 | 0.0082 | 1.00e-04 | 0.8112 | 1.4508 |
| Europe | 12 | 0.0781 | 0.0161 | 7.00e-04 | 0.7026 | 1.3182 |
| North America | 12 | 0.1776 | 0.024 | 0 | 0.8453 | 1.1665 |

*Notes on reproducibility*

All six tables and Figure S1 are regenerated by running the R script HantaV6.R on the source dataset Hanta.csv. The R pipeline outputs the same six CSV files and the figure. Numbers in Tables S1, S2, S3 are read directly from the analysis pipeline; numbers in Tables S4, S5, S6 reproduce the corresponding R outputs to 4 decimal places (minor differences in the third or fourth decimal may arise from differences in the hexagonal-grid generation between sf::st_make_grid in R and the equivalent equal-area implementation in Python used for this document; the qualitative patterns and the BH-adjusted significance pattern are identical).
