# Supplementary material for: Increasing spatial scale of analysis weakens and can mask the dilution effect for hantaviruses
Source: PLOS Glob Public Health. 2026 Sep 8;6(9):e0007045. doi: 10.1371/journal.pgph.0007045 (PMC13552722; doi:10.1371/journal.pgph.0007045)
Supplement: Supplementary file 7 [file pgph.0007045.s007.docx]

**Fig S1.** Effect of rodent species richness on hantavirus prevalence in temperate (blue, ≥23.43°N) versus tropical (red, <23.43°N) North America, across twelve spatial grains. Filled circles with black outline: BH-adjusted P < 0.05 under the quasi-binomial GLM. Empty circles: not significant. Error bars: BCa 95% confidence intervals from 1,000 bootstrap resamples of cells. Note that BCa intervals overlap zero at all grains in both subsets, so the apparent significance under the quasi-binomial test should be interpreted with caution, especially in the tropical subset (5-14 cells per grain). A small horizontal offset is applied to the two subsets to avoid marker overlap.
